# Differences and Correlations in Nutrient Intake and Hematological Markers Between Iron-Deficient and Non-Iron-Deficient Female Basketball Players: A Preliminary Study

**DOI:** 10.3390/nu18111718

**Published:** 2026-05-27

**Authors:** Kinga Piotrowska, Jakub Adamczewski, Tomasz Podgórski, Mikołaj Szymocha, Krzysztof Durkalec-Michalski

**Affiliations:** 1 Department of Sports Dietetics, Poznan University of Physical Education, 61-871 Poznan, Poland; piotrowska.kinga.pk@gmail.com (K.P.); adamczewski@awf.poznan.pl (J.A.); szymocha@awf.poznan.pl (M.S.); 2 Department of Biochemistry, Poznan University of Physical Education, 61-871 Poznan, Poland; podgorski@awf.poznan.pl; 3 Polish Society of Nutritional Sciences, 02-776 Warsaw, Poland

**Keywords:** basketball, iron deficiency, nutrient intake, females, sports nutrition

## Abstract

Background/Objectives: Iron deficiency (ID) can occur before anemia and may impair performance, recovery, and hematological function, particularly in athletes. Female basketball players may be especially vulnerable due to high training demands and sex-specific iron losses. Therefore, this study aimed to compare nutrient intake and hematological and iron status biomarkers between ID and non-ID female basketball players, and to examine diet–biomarker correlations. Methods: Twenty-four female basketball players completed the study. Athletes were stratified by ferritin, with ID defined as <30 μg/L, resulting in 12 athletes per group. Dietary intake was assessed using a 48 h food record. Energy, macronutrients, fiber, iron, calcium, folate, vitamin B_12_, and vitamin C intakes were analyzed. Blood biomarkers included red blood cells (RBCs), hemoglobin (HGB), hematocrit (HTC), mean corpuscular volume (MCV), mean corpuscular hemoglobin (MCH), mean corpuscular hemoglobin concentration (MCHC), ferritin, serum iron, transferrin, total iron-binding capacity (TIBC), and unsaturated iron-binding capacity (UIBC). Results: ID athletes had significantly lower fiber, iron, folate, and vitamin C intakes than non-ID. They also showed significantly lower HGB, HTC, MCV, MCH, and ferritin, and higher transferrin, UIBC and TIBC. Iron intake correlated positively with HGB, HTC, MCV, MCH, serum iron, and ferritin, and negatively with UIBC. Conclusions: Iron deficient female basketball players may present less favorable dietary profiles and altered hematological and iron status biomarkers. In this context, quarterly assessment of iron status biomarkers should be supported by nutrition education aimed at improving iron intake, alongside monitoring of energy and macronutrient intake in relation to training load. These approaches may help identify athletes requiring nutrition-focused support, although larger studies with longer-term dietary assessment are warranted.

## 1. Introduction

Anemia is a global health concern, with the World Health Organization estimating that its prevalence among females is approximately 30% [1]. However, iron deficiency (ID) does not always equate to anemia, as depleted iron stores affect nearly 57% of females aged 18–50 [2]. This distinction highlights that iron depletion often precedes clinically overt anemia and may be overlooked when hemoglobin (HGB) is considered in isolation. Therefore, ferritin is particularly relevant, as it reflects body iron stores and may indicate early ID even when HGB values remain within the normal range [3,4].

ID may result from various factors, including inadequate dietary intake (e.g., with low iron [Fe] intake at the forefront), and the consumption of selected inhibitors of Fe absorption (such as phytates present in plant-based foods, coffee, tea or milk ingested with meals) [5]. Furthermore, high calcium (Ca) [6] or fiber (FIB) intakes [6], intestinal diseases (including celiac disease [7] or increased intestinal permeability [8]), and intensive athletic training [9] may also contribute to this condition. Rather than reflecting a single cause, ID in athletes is usually multifactorial, resulting from the interaction between dietary intake, absorption, exercise-related losses, and individual physiological factors. Adequate Fe intake is crucial for proper human physiological functioning, and its requirements vary across different life stages and physiological states [10,11]. Moreover, Fe is a structural component of HGB and fulfills numerous biological functions: it transports oxygen, serves as a key component of various proteins and enzymes, and participates in energy pathways leading to the production of adenosine triphosphate [12,13]. Proper Fe intake is also indispensable for immune system support and tissue regeneration [10]. Accordingly, symptoms of ID are often non-specific, encompassing headaches, general fatigue, malaise, poor concentration [14], and may be misidentified as overtraining. It is crucial to emphasize that even in the absence of clinical anemia, the mere depletion of ferritin status may lead to a decline in physical capacity and performance [15,16].

Low ferritin concentrations are a common issue in sports nutrition, affecting both endurance athletes and team-sport players [17,18]. In athletic populations, iron depletion may be driven not only by insufficient dietary intake but also by exercise-related losses and impaired absorption. Athletes may lose Fe through skin diseases [19], sweating [20], micro-injuries, gastrointestinal blood loss [8], and exercise-induced hemolysis [21]. Furthermore, physical exertion stimulates the secretion of hepcidin, a regulatory hormone that limits Fe absorption [15]. Another contributing factor may be low energy availability (LEA), accompanied by inadequate Fe intake [22,23]. These mechanisms are particularly relevant in female athletes, in whom menstrual blood loss and fluctuations across the menstrual cycle may further increase susceptibility to ID [24].

Although evidence on ID in athletes is growing, female athletes remain underrepresented in sports science [25], and this sex-based data gap is especially visible in team-sports. Consequently, there is still limited sport-specific evidence regarding nutritional and hematological risk profiles in female team-sport players. This gap is important because the physiological demands of team-sports differ from those of endurance disciplines, and findings from mixed-sport or endurance populations may not be directly transferable to basketball.

Basketball represents a relevant model in this context because it is an intermittent, high-intensity team-sport characterized by a speed-strength nature of efforts, repeated accelerations, frequent changes in direction, and substantial training loads [26,27]. During a game, players engage in various activities of differing intensities and durations [27] and typically cover a distance of 5–6 km per match, with average intensity exceeding 85% of maximum heart rate [26]. Such demands may contribute to iron depletion through exercise-related losses and inflammatory regulation of Fe absorption, while high energy expenditure may exacerbate the risk of LEA and suboptimal micronutrient intake [22]. Given that these physiological demands must be supported by adequate dietary intake, proper nutrition (particularly sufficient energy [EN] and micronutrient intakes) plays a key role in effective recovery and performance in team-sports athletes [28,29]. Therefore, the primary aim of this preliminary pilot study was to examine whether EN and nutrient intakes, as well as selected blood marker concentrations differed in female basketball players depending on their iron status. This primary analysis was treated as the main hypothesis-driven comparison. We hypothesized that athletes with ID would present less favorable dietary intake profiles than their non-ID counterparts, reflected by lower intakes of EN, macronutrients, minerals and vitamins. As a secondary and exploratory aim, we examined correlations between dietary intake variables, i.e., EN, carbohydrates (CHO), proteins (PRO), fats (FAT), FIB, minerals (Fe, Ca), vitamins (C, B_12_, and folate), and blood biomarkers, i.e., red blood cells (RBCs), HGB, hematocrit (HTC), mean corpuscular volume (MCV), mean corpuscular hemoglobin (MCH), and mean corpuscular hemoglobin concentration (MCHC); and ferritin, serum iron, transferrin, total iron-binding capacity (TIBC), and unsaturated iron-binding capacity (UIBC). Within this exploratory correlation analysis, dietary Fe intake was considered the main variable of interest, with expected positive associations with ferritin, serum iron, and selected hematological outcomes, and inverse associations with iron-binding indices (transferrin, TIBC and UIBC). All remaining diet–biomarker relationships were interpreted as exploratory and hypothesis-generating, particularly considering the preliminary design.

## 2. Materials and Methods

### 2.1. Participants

Participants were recruited from leading national female basketball clubs. Female players meeting the inclusion criteria were not recruited based on known ID status before enrollment; group allocation was performed after ferritin assessment. To improve between-group comparability, non-ID athletes were randomly selected to match the ID group as closely as possible in terms of anthropometric characteristics (i.e., body height and body mass ([BM]), and training context (i.e., training and competition volume). Twenty-four female basketball players completed the study. The study was conducted in accordance with the Declaration of Helsinki (classified as: highly trained *n* = 15, elite *n* = 9) [30]. To be enrolled in the study, participants had to meet the following eligibility criteria: female sex, age 16–35 years (inclusive), at least 4 years of basketball training experience, at least 4 training sessions/week, valid medical clearance for competitive sports, good overall health, and written informed consent provided. Exclusion criteria included: recent injuries, health contraindications, and the use of ergogenic or buffering supplements (excluding continuous use of PRO, CHO, or isotonic beverages) within three months prior to enrollment. In addition, none of the participants reported the use of vitamin or mineral supplements, including iron, vitamin C, folate, or vitamin B_12_, during the period considered in the study. Ethical approval was obtained from the Bioethics Committee at Poznan University of Medical Sciences (protocol code: 404/23; date of approval 11 May 2023). The study was prospectively registered at ClinicalTrials.gov (protocol code: NCT07092930; date of registration 30 July 2025), conducted in compliance with the Declaration of Helsinki [31] and reported following CONSORT guidelines [32] (Table S1).

### 2.2. Study Design

The present study was conducted during the competitive season. Participants attended a laboratory visit during which anthropometric and body composition measurements were taken, and blood samples were collected for hematological and biochemical assessments. To standardize conditions, athletes refrained from high-intensity or prolonged exercise for 24 h before measurements. In addition, participants consumed a standardized meal 3 h prior to testing with compliance verified upon arrival [33]. Measurements were conducted during ‘non-bleeding days’ [34] of the menstrual cycle. During the laboratory visit, urine samples were also collected and analyzed using routine urinalysis (Uryxxon^®^ Relax, Macherey-Nagel, Düren, Germany) to confirm the absence of RBC, which was used as an additional control to exclude potential menstrual blood contamination before hematological and biochemical assessments.

### 2.3. Anthropometric and Body Composition Measurements

Anthropometric and body composition measurements were conducted at the beginning of the visit. Height and BM were assessed twice using a medical scale with a stadiometer (WPT 60/150 OW, Radwag^®^, Radom, Poland). Subsequently, body composition was measured using the bioelectrical impedance (101 BIVA^®^ PRO, Akern, Pisa, Italy), in accordance with the methods described elsewhere [35]. The outcomes included total body water (TBW), fat-free mass (FFM), and fat mass (FM), expressed as a percentage of BM.

### 2.4. Analyses of Dietary Records

Prior to the study, participants received standardized guidance from a certified dietitian on accurate completion of food records to improve recording quality [36]. Dietary intake was assessed using a 48 h food record, covering one training/match day and one rest day [37]. Further, dietary records were reviewed with the dietitian during the laboratory visit to confirm completeness and resolve ambiguities in reported intake [38]. Intakes were calculated for each day of the 48 h food record and then averaged across the two-day recording period for each participant (AvoDiet, JustForFood sp. z o.o., Gdańsk, Poland). Analyses included intakes of EN, CHO, PRO, FAT, FIB, minerals (Fe, Ca) and vitamins (folate, B_12_, C).

### 2.5. Hematological and Biochemical Assessments

Blood sample collection was performed using a sterile, single-use lance (Medlance^®^ Red, HTL-STREFA, Chorzów, Poland) from the finger of the non-dominant hand by experienced personnel following procedures described previously [33]. Using <10 µL capillary blood collected into an EDTA-tube, the following indicators were assessed: RBC, HGB, HTC, MCV, MCH and MCHC on a 20-parameter automated hematology analyzer (Mythic 18^®^, Orphée, Geneva, Switzerland). Approximately 300 µL of capillary blood was collected into serum tubes and allowed to clot at room temperature. Fully clotted samples were then centrifuged to obtain serum [39] and iron status markers were immediately quantified (Accent 220S, Cormay, Łomianki, Poland). Serum iron and UIBC concentrations were measured colorimetrically (Accent-200 Ferrum, Cormay, Łomianki, Poland; sensitivity test: 4.1 μg/dL; Accent-200 UIBC, Cormay, Łomianki, Poland; sensitivity test: 20 μg/mL). Transferrin (Accent-200 Transferrin, Cormay, Łomianki, Poland; sensitivity test: 0.076 g/L) and ferritin (Accent-200 Ferritin, Cormay, Łomianki, Poland; sensitivity test: 9.1 ng/mL) were determined by turbidimetry based on specific antigen–antibody reactions. TIBC was calculated by summing measured serum iron and UIBC.

### 2.6. Participants Stratification

To enable a more in-depth evaluation of dietary intake and iron status biomarkers, participants were stratified by ferritin concentration. ID was defined as serum ferritin <30 μg/L, whereas participants with ferritin ≥30 μg/L were classified as non-iron-deficient (non-ID) [40,41]. This threshold was selected because of its widespread recognition in sports medicine and exercise-nutrition research. Moreover, it allows it to indicate depleted or insufficient iron stores in athletes, even when HGB values remain within the normal range. In total, 24 athletes were included and allocated evenly to two groups (ID, *n* = 12 [highly trained *n* = 9, elite *n* = 3]; non-ID, *n* = 12 [highly trained *n* = 6, elite *n* = 6]).

### 2.7. Sample Size Calculation

Given the pilot nature of the study, an *a priori* sample size calculation was performed for the primary comparison between ID and non-ID using a two-tailed independent-samples *t*-test (G*Power, version 3.1.9.7., Heinrich Heine University Düsseldorf, Düsseldorf, Germany) [42]. The calculation was based on HGB [40], and the effect size was derived from previously reported differences between female athletes with and without ID [41]. HGB was selected because it is a clinically relevant and widely reported hematological marker related to iron status, with available comparative data in female athletes. Since serum ferritin was used as the stratification variable in the present study, HGB provided an independent and clinically interpretable basis for the sample size calculation. This choice was not intended to imply that HGB is more sensitive than serum ferritin for detecting early ID. Assuming α of 0.05, power of 0.80 [43], and an allocation ratio of 1:1, the required final analytic sample was estimated at 24 athletes (12 per group).

### 2.8. Statistical Analyses

Prior to the analyses, assumptions were evaluated within each group (Statistica, version 13.3, TIBCO Software Inc., Palo Alto, CA, USA) [44]. Normality was assessed using the Shapiro–Wilk test. When data were not normally distributed, the Mann–Whitney U test was applied. For normally distributed variables, homogeneity of variances was examined using Levene’s test; if this assumption was met, an independent-samples *t*-test was used, and if not, Welch’s *t*-test was applied. Results are presented as mean ± standard deviation (SD) and 95% confidence intervals (CI). Exploratory correlations were assessed across the entire sample using Pearson’s *r* for normally distributed variables and Spearman’s *r* otherwise. Correlation strength was interpreted as *negligible* (0.00–0.30), *low* (0.31–0.50), *moderate* (0.51–0.70), *high* (0.71–0.90) or *very high* (0.91–1.00) [44], across the full range of −1 to +1. Statistical significance was set at α < 0.05.

## 3. Results

### 3.1. Basic Characteristics of Participants

No significant differences between non-ID and ID were observed in age (21.2 ± 4.6 vs. 19.8 ± 3.1 years), basketball experience (11.2 ± 3.0 vs. 10.9 ± 3.2 years), training volume (12.5 ± 0.9 vs. 12.2 ± 0.6 h/week) or competition volume (1.2 ± 0.4 vs. 1.1 ± 0.3 matches/week).

### 3.2. Anthropometric and Body Composition Outcomes

No significant differences between non-ID and ID were observed in body height, BM, TBW, FFM and FM (Table 1).

### 3.3. Dietary Records Outcomes

Significant differences between non-ID and ID were observed in FIB, Fe, folate and vitamin C intakes. No significant differences between non-ID and ID were observed in EN, PRO, FAT, CHO, Ca and vitamin B_12_ intakes (Table 2).

### 3.4. Hematological and Biochemical Outcomes

Significant differences between non-ID and ID were observed in HGB, HTC, MCH, MCV, ferritin, transferrin, UIBC and TIBC. No significant differences between non-ID and ID were observed in RBC, MCHC, and serum iron (Table 3).

### 3.5. Correlations

**Figure 1:**
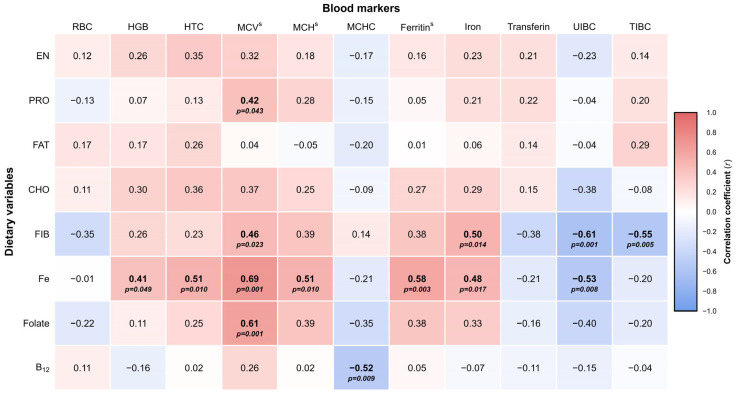
Correlation outcomes. ^S^ analyzed using Spearman’s rank correlation coefficient. Abbreviations: B_12_, vitamin B_12_; CHO, carbohydrates; EN, energy; FAT, fats; Fe, iron; FIB, fiber; HGB, hemoglobin; HTC, hematocrit; MCH, mean corpuscular hemoglobin; MCHC, mean corpuscular hemoglobin concentration; MCV, mean corpuscular volume; *p*, probability value; PRO, proteins; RBC, red blood cell; TIBC, total iron-binding capacity; UIBC, unsaturated iron-binding capacity. Note: Correlation coefficients presented in bold indicate statistically significant correlations (*p* < 0.05).

Significant positive correlations were found for PRO–MCV, FIB–MCV, FIB–serum iron, Fe–HGB, and Fe–serum iron (*low*); Fe–MCV, Fe–HTC, Fe–MCH, Fe–ferritin, and folate–MCV (*moderate*). Significant negative correlations were found for FIB–UIBC, FIB–TIBC, Fe–UIBC, and vitamin B_12_–MCHC (*moderate*). No significant correlations were found for the remaining variables (Figure 1).

## 4. Discussion

### 4.1. Main Findings

The primary aim of this preliminary study was to examine whether EN and nutrient intakes differ in female basketball players depending on their iron status.

Analysis of the participants’ characteristics revealed no significant differences in BM, body composition or training/competition volume between the groups. This stability is a positive finding, as it indicates a high degree of homogeneity within the study population and may suggest that the observed biochemical differences result from specific dietary factors.

The results partially confirmed the primary hypothesis, particularly regarding differences in the intake of selected minerals and vitamins associated with maintaining adequate ferritin status. The most significant finding of this study is that female basketball players with ID had significantly lower dietary Fe intake than the non-ID group. Participants in the ID group met only approximately 70% of the recommended daily allowance for females [29]. Similar observations have been reported in other athletic populations (such as football [45,46]), suggesting that inadequate Fe intake may represent a broader nutritional concern among athletes [16]. Consistent with these findings, a high prevalence of ID has been reported among sub-elite female endurance athletes [41]; however, this study also pointed toward inadequate dietary intake as a primary factor. The absence of detailed dietary logs limited the ability to precisely quantify nutrient intake levels. In our study, differences in vitamin intakes were also noted between groups, particularly for vitamin C and folate. The lower vitamin C intake revealed in the ID group appears especially relevant, as vitamin C is the most potent promoter of non-heme Fe absorption and may therefore further reduce Fe bioavailability [5,47]. Accordingly, ensuring an adequate intake may help optimize the absorption of dietary Fe in athletes [48]. Additionally, significantly lower folate intake observed in the ID group may further compromise hematological health, as folates are essential for erythropoiesis and DNA synthesis [49]. The *moderate* positive correlation found between folate intake and MCV suggests that suboptimal supply of this vitamin may be relevant to hematological monitoring in female athletes, given the role of folate in erythropoiesis and DNA synthesis [50,51]. However, because fatigue and recovery were not directly assessed in the present study, these implications should be interpreted cautiously. Consequently, considering folate alongside Fe intake may be useful for supporting nutritional strategies aimed at maintaining an adequate RBC profile in female athletes. While we also hypothesized lower EN intake in athletes with ID, no significant differences were observed between groups. Nevertheless, the observed difference of approximately 250 kcal/day, although only hypothetically, may suggest a risk of LEA within the ID group [22].

The analysis of hematological and biochemical indicators carried out in our study further emphasizes the impact of these dietary patterns. Athletes with ID exhibited significantly lower HGB, HTC, MCV, and MCH. However, RBC remained unaffected despite depleted ferritin status, which is consistent with earlier research [52]. This is particularly relevant because low ferritin concentrations have been associated in the literature with outcomes relevant to athletic performance and impaired recovery [53]. However, as no direct performance or recovery measures were collected in the present study, our findings should be interpreted as relevant to athlete health and monitoring rather than as evidence of impaired performance. The concomitant increase in transferrin, TIBC, and UIBC observed in the ID group represents a characteristic physiological response [54] aimed at maximizing the body’s Fe transport capacity. In accordance with clinical guidelines [55], such elevations serve as a critical compensatory mechanism to counteract low ferritin status by enhancing the efficiency of serum iron mobilization.

Regarding the secondary aim, significant correlations between dietary variables and blood markers were found. As predicted, Fe intake was positively associated with ferritin and HGB, while iron-binding indices (TIBC, UIBC, and transferrin) showed an inverse relation. Furthermore, an interesting result was the significantly higher FIB intake in the non-ID group compared to the ID group. Although FIB may negatively influence Fe absorption depending on the dietary matrix, its higher intake in non-ID group may primarily reflect better overall diet quality rather than a direct beneficial effect of FIB on iron status. A diet richer in whole grains, vegetables, and other minimally processed foods may simultaneously provide greater amounts of Fe, folate, and vitamin C [56]. This interpretation is further supported by *moderate* positive correlations between FIB–serum iron, and FIB–MCV; however, these associations should be interpreted cautiously given the preliminary design. Despite no significant differences in Ca intake between the groups, its potential role as a potential inhibitor of Fe absorption remains relevant from a practical perspective. The effect of Ca on Fe absorption depends on meal composition, and Ca may inhibit both heme and non-heme Fe absorption when consumed within the same meal [6]. However, because meal timing and food combinations were not directly analyzed in the present study, separating Ca-rich products from Fe-rich meals, particularly around the post-exercise recovery period, should be considered a practical hypothesis rather than a direct conclusion from our findings. Future studies should examine meal-level nutrient interactions to better determine whether Ca timing influences Fe bioavailability in female basketball players. Focusing on the physical exertion performed by the team-sport athletes, professional basketball practice may pose additional challenges to ferritin homeostasis, as the high intensity and volume of training and matches can stimulate post-exercise inflammation [26]. This triggers the release of hepcidin, a hormone that inhibits Fe absorption in the intestines for several hours following activity. In combination with inadequate dietary Fe intake, these mechanisms may lead to the depletion of ferritin status and disturbances in hematological indicators [57,58]. Therefore, for the prevention and management of ID in this group, it is suggested that dietary patterns be strategically structured to optimize Fe bioavailability. Specifically, Fe-rich meals should be consumed away from training sessions, ideally in the morning or several hours post-exercise to avoid the inhibitory effects of exercise-induced hepcidin. Furthermore, these meals could be composed of potent absorption enhancers, such as vitamin C and folate, while avoiding simultaneous consumption of inhibitors like Ca or polyphenols.

### 4.2. Study Limitations

Several limitations should be considered when interpreting the results of this study. Firstly, this was a preliminary study with a relatively small sample size, which limits the statistical power and generalizability of the findings. In addition, the cross-sectional design does not allow causal inference; therefore, the observed dietary and hematological differences should be interpreted as associations rather than evidence of cause–effect relationships. The scope and design of this preliminary study were not intended to assess the overall prevalence of ID among female basketball players. The research focused specifically on identifying and analyzing the dietary and hematological differences between ID and non-ID groups.

A further limitation may be the lack of full standardization of blood collection relative to the specific phases of the participants’ menstrual cycles. Nevertheless, menstrual cycle status was monitored using a calendar-based counting method [59]. Moreover, the study was conducted in naturally menstruating females [60] during ‘non-bleeding days’ [34]. As an additional control, urine samples were collected during the visit and analyzed using routine urinalysis, including the assessment of luteinizing hormone [59]. Notably, no RBCs were detected in the urine samples obtained from the athletes during the testing. As menstrual blood contamination is considered a potential source of blood/RBC findings in urinalysis, the absence of RBCs may provide additional support that sample collection did not coincide with menstrual bleeding [61]. Furthermore, from the perspective of sports physiology, current evidence indicates that the menstrual cycle phase does not exert a significant impact on athletic performance or ferritin status [62,63].

Additionally, dietary intake was assessed using a 48 h food record, which may not fully capture habitual long-term nutritional patterns, particularly for micronutrients such as iron, folate, vitamin C, and vitamin B_12_. Nevertheless, this method has been previously applied in our team’s research involving athletes [37]. To mitigate potential reporting errors and improve data quality, all participants received standardized instructions from a certified dietitian before completing the food record and subsequently underwent a follow-up consultation to verify the records and clarify any ambiguities. Moreover, the recorded diets appeared relatively consistent across the two assessment days in terms of food choices and meal structure; however, this does not replace longer-term dietary monitoring. Therefore, dietary differences observed in the present study should be interpreted cautiously and regarded as reflective of intake during the recorded period rather than stable habitual intake.

Although a complete blood count was performed, including white blood cell, and no elevated values were observed, C-reactive protein or other specific inflammatory markers were not assessed. Therefore, despite the absence of hematological signs suggestive of overt inflammation or infection, the potential influence of subclinical inflammation on ferritin concentrations cannot be fully excluded, as ferritin is an acute-phase reactant.

Finally, direct performance or recovery outcomes were not measured; therefore, the practical implications of the observed nutritional and hematological profiles should be interpreted in relation to athlete health monitoring rather than demonstrated changes in sport performance.

### 4.3. Study Strengths

The study has several important strengths that enhance the robustness and interpretability of the findings. First, the research was conducted in a homogeneous group of highly trained female basketball players, which allowed for a precise characterization of nutrient intake and ID within a narrowly defined athletic population. Moreover, the novelty of this study can be considered at three complementary levels: sex, sport discipline, and training status. At the sex-specific level, the study focuses on female athletes, a population for whom iron status is particularly relevant due to sex-related physiological factors and an increased risk of ID. At the sport-specific level, the study addresses basketball, a high-intensity intermittent team sport with distinct physiological and nutritional demands. At the training-status level, the study provides evidence from highly trained athletes, in whom dietary adequacy and iron status are especially relevant because of high training loads and competition demands. Therefore, the novelty of the present study lies not in showing that ID may coexist with less favorable dietary intake, but in providing a sex-, sport-, and training status-specific assessment of nutrient intake and iron-status biomarkers in highly trained female basketball players.

Second, the study employed a comprehensive assessment of iron status by analyzing a broad panel of hematological and iron-related biomarkers, including ferritin, serum iron, transferrin, TIBC, UIBC, and RBC indices. This approach provided a more reliable diagnostic representation of iron status than single-marker assessment and strengthened the interpretation of the dietary findings.

Third, the evaluation of associations between dietary components and biochemical variables offers deeper insight into dietary factors associated with iron status in highly trained female basketball players. This is particularly important because iron status is influenced by multiple nutritional and physiological factors, and its interpretation may be limited when dietary intake and biochemical markers are considered separately.

Fourth, from an academic perspective, the study provides a focused basis for further research on the relationship between dietary adequacy and ID risk in female athletes. In particular, the findings help identify dietary variables and iron-related biomarkers that warrant further investigation in longitudinal and intervention studies. Thus, the academic implication of this study lies in generating more specific research questions regarding whether less favorable dietary intake precedes, accompanies, or follows alterations in iron status.

### 4.4. Practical Applications

**Figure 2:**
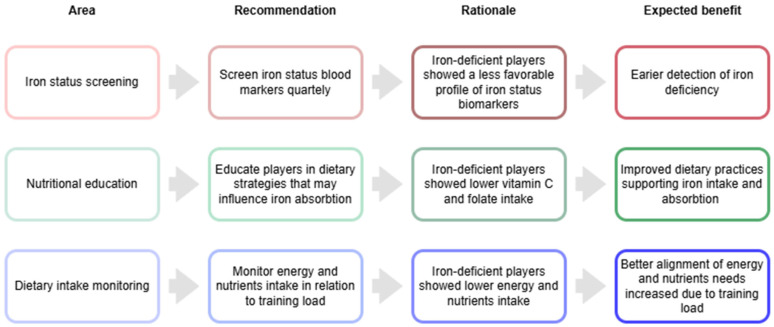
Practical recommendations for supporting iron status in female basketball players. Note: The gradual increase in color intensity from left to right illustrates the progression from the area to the expected benefit and does not indicate priority or strength of evidence.

Based on the present findings, strategies to support iron status in female basketball players were formulated (Figure 2). In female team-sport athletes, such monitoring should be considered as part of a broader performance-oriented framework, as sport-specific performance depends on multiple interacting factors, including nutritional status, hematological profile, neuromuscular readiness, reaction time, and movement speed [64]. To enhance clarity and applicability, they were organized into key areas of intervention, with each strategy accompanied by its rationale and expected benefit. This structure was intended to facilitate translation of the study findings into targeted screening, nutritional, and monitoring approaches. Therefore, three main practical recommendations were formulated: (1) quarterly assessment of iron status blood markers, which is consistent with previous recommendations for regular screening in female team-sport athletes [18] and is further supported by the present findings; (2) education of players on dietary strategies that may influence Fe absorption; and (3) monitoring energy and macronutrient intake in relation to training load.

## 5. Conclusions

The findings of this preliminary study indicate that iron deficiency among highly trained female basketball players was associated with less favorable dietary intake profiles and selected hematological and iron-related biomarkers. The results highlight that overall diet quality, including adequate intakes of iron, folate, vitamin B_12_, vitamin C and fiber, may be relevant for maintaining iron homeostasis in female basketball players. However, these findings should be interpreted as preliminary and hypothesis-generating. Larger longitudinal studies including longer dietary monitoring, inflammatory markers, menstrual status assessment, and direct performance and recovery outcomes are needed to confirm these observations and clarify their practical significance.

## Figures and Tables

**Table 1:** Anthropometric and body composition outcomes.

Indicator	Unit	Non-ID	ID	*p*
Body height	cm	178.7 ± 7.6 (173.9–183.5)	178.4 ± 9.3 (172.5–184.3)	0.943
Body mass	kg	72.4 ± 8.5 (67.0–77.8)	72.6 ± 8.3 (67.4–77.9)	0.940
Total body water	%	53.9 ± 3.2 (51.9–55.9)	52.2 ± 3.6 (49.9–54.5)	0.244
Fat-free mass	%	73.0 ± 4.7 (70.1–76.0)	71.3 ± 4.9 (68.2–74.4)	0.372
Fat mass	%	27.0 ± 4.7 (24.0–29.9)	28.7 ± 4.9 (25.6–31.9)	0.364

Abbreviations: ID, iron-deficient; non-ID, non-iron-deficient; *p*, probability value. Note: Values are mean ± standard deviation; square brackets indicate 95% CI (lower–upper limits).

**Table 2:** Dietary records outcomes.

Indicator	Unit	Non-ID	ID	*p*
Energy intake	kcal/day	2615 ± 417 (2351–2881)	2370 ± 363 (2140–2601)	0.138
	kcal/kg/day	36.5 ± 6.6 (32.3–40.7)	33.3 ± 7.5 (28.5–38.1)	0.279
Proteins	g/day	118 ± 23 (104–133)	106 ± 26 (90–123)	0.240
	g/kg/day	1.7 ± 0.4 (1.4–1.9)	1.5 ± 0.5 (1.2–1.8)	0.409
Fats	g/day	91 ± 16 (81–102)	88 ± 24 (74–104)	0.780
	g/kg/day	1.3 ± 0.3 (1.1–1.5)	1.2 ± 0.4 (1.0–1.5)	0.792
Carbohydrates	g/day	341 ± 75 (293–388)	294 ± 54 (260–328)	0.061
	g/kg/day	4.7 ± 1.0 (4.1–5.4)	4.1 ± 1.0 (3.5–4.8)	0.156
Fiber	g/day	29.2 ± 7.5 (24.4–34.0)	21.8 ± 6.6 (17.6–26.0)	0.019 *
Calcium	mg/day	843 ± 337 (629–1057)	661 ± 266 (492–830)	0.156
Iron	mg/day	16.3 ± 2.6 (14.7–17.9)	12.7 ± 2.4 (11.1–14.2)	0.002 *
Folate	μg/day	499 ± 105 (432–565)	357 ± 108 (288–426)	0.004 *
Vitamin B_12_	μg/day	3.8 ± 1.5 (2.9–4.8)	3.2 ± 1.7 (2.1–4.3)	0.329
Vitamin C	mg/day	188 ± 84 (135–242)	113 ± 97 (51–174)	0.012 *

* indicates significant between-group difference (*p* < 0.05). Abbreviations: ID, iron-deficient; non-ID, non-iron-deficient; *p*, probability value. Note: Values are mean ± standard deviation; square brackets indicate 95% CI (lower–upper limits).

**Table 3:** Hematological and biochemical outcomes.

Indicator	Unit	Non-ID	ID	*p*
Red blood cells	10^12^/L	4.6 ± 0.3 (4.4–4.8)	4.6 ± 0.4 (4.4–4.8)	0.892
Hemoglobin	mmol/L	8.3 ± 0.5 (8.1–8.6)	7.7 ± 0.7 (7.3–8.1)	0.013 *
Hematocrit	L/L	0.399 ± 0.025 (0.383–0.415)	0.369 ± 0.025 (0.353–0.385)	0.009 *
Mean corpuscular hemoglobin	fmol	1.82 ± 0.10 (1.76–1.89)	1.69 ± 0.19 (1.57–1.81)	0.046 *
Mean corpuscular volume	fL	87.3 ± 4.2 (84.6–90.0)	80.8 ± 8.1 (75.6–85.9)	0.022 *
Mean corpuscular hemoglobin concentration	mmol/L	20.9 ± 0.5 (20.6–21.2)	20.9 ± 0.7 (20.4–21.3)	0.855
Ferritin	μg/dL	57.3 ± 17.9 (46.0–68.7)	21.8 ± 3.6 (19.6–24.1)	0.001 *
Iron	μg/dL	95.2 ± 34.6 (73.2–117.2)	69.2 ± 29.8 (50.3–88.2)	0.062
Transferrin	g/L	2.9 ± 0.6 (2.5–3.2)	3.5 ± 0.6 (3.1–3.9)	0.011 *
Unsaturated iron-binding capacity	μg/dL	233 ± 101 (169–298)	368 ± 71 (323–413)	0.001 *
Total iron-binding capacity	μg/dL	353 ± 82 (301–405)	437 ± 66 (395–479)	0.011 *

* indicates significant between-group difference (*p* < 0.05). Abbreviations: ID, iron-deficient; non-ID, non-iron-deficient; *p*, probability value. Note: Values are mean ± standard deviation; square brackets indicate 95% CI (lower–upper limits).

## Data Availability

The original contributions presented in the study are included in the article. Further inquiries can be directed at the corresponding author.

## Supplementary Materials

The following supporting information can be downloaded at: https://www.mdpi.com/article/10.3390/nu18111718/s1, Table S1: CONSORT 2025 checklist.

## Abbreviations

The following abbreviations are used in this manuscript:

BM	Body mass
Ca	Calcium
CHO	Carbohydrates
EN	Energy
FAT	Fats
Fe	Iron (dietary)
FFM	Fat-free mass
FIB	Fiber
FM	Fat mass
HGB	Hemoglobin
HTC	Hematocrit
ID	Iron-deficient
LEA	Low energy availability
MCH	Mean corpuscular hemoglobin
MCHC	Mean corpuscular hemoglobin concentration
MCV	Mean corpuscular volume
Non-ID	Non-iron-deficient
PRO	Proteins
RBC	Red blood cell
TBW	Total body water
TIBC	Total iron-binding capacity
UIBC	Unsaturated iron-binding capacity
