# Supplementary material for: Differences and Correlations in Nutrient Intake and Hematological Markers Between Iron-Deficient and Non-Iron-Deficient Female Basketball Players: A Preliminary Study
Source: Nutrients. 2026 May 27;18(11):1718. doi: 10.3390/nu18111718 (PMC13259344; doi:10.3390/nu18111718)
Supplement: Supplementary file 1 [file nutrients-18-01718-s001.zip › nutrients-4321971-supplementary.pdf]

Table S1. CONSORT 2025 checklist

Section/topic	No	CONSORT 2025 checklist item description	Reported on page no.
**Title and abstract**			
Title and structured abstract	1a	Identification as a randomised trial	Not applicable.
	1b	Structured summary of the trial design, methods, results, and conclusions	Page 1, Abstract.
**Open science**			
Trial registration	2	Name of trial registry, identifying number (with URL) and date of registration	Page 3, Section 2.1. Participants. ClinicalTrials.gov identifier reported: NCT07092930.
Protocol and statistical analysis plan	3	Where the trial protocol and statistical analysis plan can be accessed	Not reported.
Data sharing	4	Where and how the individual de-identified participant data (including data dictionary), statistical code and any other materials can be accessed	Page 11, Data Availability Statement.
Funding and conflicts of interest	5a	Sources of funding and other support (eg, supply of drugs), and role of funders in the design, conduct, analysis and reporting of the trial	Page 10, Funding. Funding source reported.
	5b	Financial and other conflicts of interest of the manuscript authors	Page 11, Conflicts of Interest.
**Introduction**			
Background and rationale	6	Scientific background and rationale	Pages 1–3, Introduction.
Objectives	7	Specific objectives related to benefits and harms	Pages 2–3, Introduction. Primary and secondary aims and hypotheses reported; harms are not applicable because no intervention was administered.
**Methods**			
Patient and public involvement	8	Details of patient or public involvement in the design, conduct and reporting of the trial	Not applicable.
Trial design	9	Description of trial design including type of trial (eg, parallel group, crossover), allocation ratio, and framework (eg, superiority, equivalence, non-inferiority, exploratory)	Pages 3–4, Sections 2.2. Study Design and 2.6. Participants Stratification. Preliminary pilot study with ferritin-based stratification into ID and non-ID groups; 12 athletes per group.
Changes to trial protocol	10	Important changes to the trial after it commenced including any outcomes or analyses that were not prespecified, with reason	Not applicable.
Trial setting	11	Settings (eg, community, hospital) and locations (eg, countries, sites) where the trial was conducted	Page 3, Section 2.2. Study Design. Laboratory visit reported; institutional affiliation and ethics approval reported on pages 1 and 11.
Eligibility criteria	12a	Eligibility criteria for participants	Page 3, Section 2.1. Participants.
	12b	If applicable, eligibility criteria for sites and for individuals delivering the interventions (eg, surgeons, physiotherapists)	Not applicable.
Intervention and comparator	13	Intervention and comparator with sufficient details to allow replication. If relevant, where additional materials describing the intervention and comparator (eg, intervention manual) can be accessed	Not applicable.

Outcomes	14	Prespecified primary and secondary outcomes, including the specific measurement variable (eg, systolic blood pressure), analysis metric (eg, change from baseline, final value, time to event), method of aggregation (eg, median, proportion), and time point for each outcome	Pages 3–4, Sections 2.3–2.6. Anthropometric/body composition outcomes, dietary intake variables, hematological markers, biochemical iron-status markers, and ferritin-based stratification reported.
Harms	15	How harms were defined and assessed (eg, systematically, non-systematically)	Not applicable.
Sample size	16a	How sample size was determined, including all assumptions supporting the sample size calculation	Page 4, Section 2.7. Sample Size Calculation.
	16b	Explanation of any interim analyses and stopping guidelines	Not applicable.
Randomisation:			
Sequence generation	17a	Who generated the random allocation sequence and the method used	Not applicable.
	17b	Type of randomisation and details of any restriction (eg, stratification, blocking and block size)	Not applicable.
			**Reported on page no.**
Allocation concealment mechanism	18	Mechanism used to implement the random allocation sequence (eg, central computer/telephone; sequentially numbered, opaque, sealed containers), describing any steps to conceal the sequence until interventions were assigned	Not applicable.
Implementation	19	Whether the personnel who enrolled and those who assigned participants to the interventions had access to the random allocation sequence	Not applicable.
Blinding	20a	Who was blinded after assignment to interventions (eg, participants, care providers, outcome assessors, data analysts)	Not applicable.
	20b	If blinded, how blinding was achieved and description of the similarity of interventions	Not applicable.
Statistical methods	21a	Statistical methods used to compare groups for primary and secondary outcomes, including harms	Pages 4–5, Section 2.8. Statistical Analyses.
	21b	Definition of who is included in each analysis (eg, all randomised participants), and in which group	Page 4, Section 2.6. Participants Stratification; page 5, Section 3.1. Basic Characteristics of Participants. Twenty-four athletes were included, with 12 athletes in each group.
	21c	How missing data were handled in the analysis	Not applicable.
	21d	Methods for any additional analyses (eg, subgroup and sensitivity analyses), distinguishing prespecified from post hoc	Pages 4–5, Section 2.8. Statistical Analyses. Exploratory correlation analyses reported; results presented on page 7, Section 3.5 and Figure 1.
**Results**			
Participant flow, including flow diagram	22a	For each group, the numbers of participants who were randomly assigned, received intended intervention, and were analysed for the primary outcome	Not applicable.
	22b	For each group, losses and exclusions after randomisation, together with reasons	Not applicable.
Recruitment	23a	Dates defining the periods of recruitment and follow-up for outcomes of benefits and harms	Not applicable.
	23b	If relevant, why the trial ended or was stopped	Not applicable.
Intervention and comparator delivery	24a	Intervention and comparator as they were actually administered (eg, where appropriate, who delivered the intervention/comparator, how participants adhered, whether they were delivered as intended (fidelity))	Not applicable.

	24b	Concomitant care received during the trial for each group	Not applicable.
Baseline data	25	A table showing baseline demographic and clinical characteristics for each group	Page 5, Section 3.1. Basic Characteristics of Participants; page 5, Table 1. Anthropometric and body composition outcomes.
Numbers analysed, outcomes and estimation	26	For each primary and secondary outcome, by group:<br>● the number of participants included in the analysis<br>● the number of participants with available data at the outcome time point<br>● result for each group, and the estimated effect size and its precision (such as 95% confidence interval)<br>● for binary outcomes, presentation of both absolute and relative effect size	Pages 5–7, Sections 3.2–3.5; Tables 1–3 and Figure 1. Group results, p-values, and 95% confidence intervals for significant between-group differences are reported.
Harms	27	All harms or unintended events in each group	Not applicable.
Ancillary analyses	28	Any other analyses performed, including subgroup and sensitivity analyses, distinguishing pre-specified from post hoc	Page 7, Section 3.5. Correlations; Figure 1. Exploratory diet–biomarker correlation analyses reported.
**Discussion**			
Interpretation	29	Interpretation consistent with results, balancing benefits and harms, and considering other relevant evidence	Pages 7–10, Section 4. Discussion; page 10, Section 5. Conclusions.
Limitations	30	Trial limitations, addressing sources of potential bias, imprecision, generalisability, and, if relevant, multiplicity of analyses	Page 9, Section 4.2. Study Limitations and Strengths.
